# Supplementary material for: Sequence heterochrony led to a gain of functionality in an immature stage of the central complex: A fly–beetle insight
Source: PLoS Biol. 2020 Oct 26;18(10):e3000881. doi: 10.1371/journal.pbio.3000881 (PMC7644108; doi:10.1371/journal.pbio.3000881)
Supplement: S1 Text — (PDF) [file pbio.3000881.s001.pdf]

Supporting Results

Mapping of Rx-positive cell groups to known lineages of the insect adult brain

We aimed at determining to which previously described lineages the Rx-positive cells belonged. The lineages in *Drosophila* had been described as the published atlas [1,2]. We reassigned them in the *Drosophila* brain and transferred *Drosophila* knowledge to the *Tribolium* brain. Assignments of conserved Rx expressing cell groups in the cell body rind in both species' brains were based on two aspects. First, synapsin staining revealed common synapse-rich neuropils as well as synapse-absent tracts and fascicles that can be homologized between the two species. With this, domains of the *Tribolium* brain could be linked to domains and known lineages in *Drosophila*. Second, since Rx-positive lineages were defined by stereotypical projections, an additional antibody staining against GFP in the characterized Rx transgenic lines (S2 and S3 Figs) revealed some lineage-typical projections. Therefore, projections helped in some cases to verify lineage identity beyond cell body position. However, for most lineages, projections were not distinguishable. We identified eleven lineages in both species that cover most of the Rx expressing cell groups in the adult brain (DALcl1/2, DALl1/2, DALv1/3, DPLal1-3, DPLc, DAMv1/2, DM1 (DPMm1), DM2/3 (DPMpm1/2), DM4 (CM4), DM5/6 (CM1/3), CP2/3 (DL1/2, S4 Fig, S1 Table): In the *Tribolium* brain, all n-ventral lineages were not marked by projections through our transgenic line. They have been identified due to the basic anatomical position of the cell bodies that was very similar to Rx expressing lineages in the *Drosophila* brain. In the *Drosophila* Rx-GFP line all n-ventral lineages were – if at all – only faintly marked by projections. Visible were projections of the lineage group DALc1/2 that projected n-posterior to the peduncle into the central complex, the likely dorsal tract of the DPLal2/3 lineage that projected into the superior lateral protocerebrum, the short projection of the DPLc1 sublineage and the dorso-medial projection of the DAMv1/2 lineage into the superior medial protocerebrum. In the n-dorsal fraction, both projections of hemilineages of the CP2/3 lineage were visible in both species, one reaching n-anterior over the peduncle and projecting into the superior medial protocerebrum, one starting n-posterior of the peduncle and projecting n-ventro-anterior to it. With the available

tools, we could not determine homology of these lineages further. To verify this tentative lineage identification, based mostly on cell body location, specific transgenic lines need to be generated and subsequent antibody stainings need to be performed, particularly in *Tribolium*, to further reveal the characteristic projection patterns of each lineage.

Description of Rx-positive subgroups of DM1-4 lineages in *Tribolium* and *Drosophila*

In addition to the general descriptions of cell body location and projections on a lineage level (Fig 2-3, S4 Figure), DM1-4 lineages were previously divided into sub-groups and single tracts [2]. We wanted to describe which of those sub-groups and tracts are visible in the *Drosophila* adult brain and describe analogous sub-groups and tracts in *Tribolium*. These groups were differently marked in the imaging lines in both species due to the different design of the transgenic lines (see S2 and S3 Figs). Note that the projections of individual tracts or neurons in the respective neuropils were hard to distinguish because a high number of cells were marked.

In *Drosophila*, the DM4 Rx expressing cell group consisted of three subgroups, one localized n-anterior, and two n-posterior to the lateral tip of the PB. They projected axons to form a common projection as part of the MEF which bifurcated near the midline, where parts went into a n-ventral midline crossing projection n-ventral to the whole CX. This projection might be partially shared by the upper intermediate tract of CM3 or the dorsal tract of CM1 [2]. The other part projected mainly into the fan-shaped body (FB; 'intermediate tract; [2]). The DM3 Rx expressing group consisted of two groups, one more n-anterior, one n-posterior to the lateral side of the PB. The group's axons formed parts of the dorso-lateral root of the fan-shaped body (dlrFB) together with DM2 in the 'anterior-ventral tract' [2]. Parts of these cells' axons projected into the n-dorsal plexus (also FBppl, see e.g. [3]), while substantial parts went in a more n-ventro-posterior part together with DM4. DM2 consisted of three groups, two n-anterior (one of which is more n-dorsal), one n-posterior to the PB. They projected together into the n-dorsal plexus of FB ('anterior-ventral tract'; [2], slightly more n-dorsal than DM3. The projection bifurcated, one more n-anterior, one more n-posterior. The DM1 group consisted of three subgroups, all n-anterior to the protocerebral bridge. One more n-ventral

and slightly more lateral, two were more n-dorsal, of which one was n-anterior to the other. The n-ventral group formed a separate more lateral projection (potentially the 'anterior descending tract'; [2]) in comparison to the common projection of the other group ('anterior-ventral tract'; [2]).

In *Tribolium*, DM4 consisted of two groups localized n-anterior to the PB tip and one n-posterior to the PB tip. The bifurcation of the tract from both groups was similar to *Drosophila*, and they thus could also share a projection with a CM3 tract. A division into an n-anterior and n-posterior part was similar to *Drosophila*. A third group present in *Drosophila* was not marked or was not present in *Tribolium*. DM3 consisted of two main groups, one more n-anterior to the PB, one n-posterior to the PB, an arrangement similar to *Drosophila*. They projected together with DM2 and 1 into the n-dorsal fraction of the FB, while sharing the dlrFB tunnel with DM3, with projections very similar to *Drosophila*. Cell bodies of DM2 were difficult to visualize but were slightly more medial to the DM3 belonging group. Hence approximate position was similar, but a subdivision in groups was hardly possible. Cell bodies of DM1 were sparse, with some n-anterior and some n-posterior to the protocerebral bridge, like *Drosophila* without a subdivision into groups possible. The projection into the FB was very similar. Note that, in general, cell groups of DM1-3 were n-dorsal, and not like in *Drosophila* n-anterior to the PB.

References

1. Lovick JK, Ngo KT, Omoto JJ, Wong DC, Nguyen JD, Hartenstein V. Postembryonic lineages of the Drosophila brain: I. Development of the lineage-associated fiber tracts. Dev Biol. 2013;384: 228–257. doi:10.1016/j.ydbio.2013.07.008

2. Wong DC, Lovick JK, Ngo KT, Borisuthirattana W, Omoto JJ, Hartenstein V. Postembryonic lineages of the Drosophila brain: II. Identification of lineage projection patterns based on MARCM clones. Dev Biol. 2013;384: 258–289. doi:10.1016/j.ydbio.2013.07.009

3. Riebli N, Viktorin G, Reichert H. Early-born neurons in type II neuroblast lineages establish a larval primordium and integrate into adult circuitry during central complex development in Drosophila. Neural Dev. 2013;8: 6. doi:10.1186/1749-8104-8-6
