# Supplementary material for: Sequence heterochrony led to a gain of functionality in an immature stage of the central complex: A fly–beetle insight
Source: PLoS Biol. 2020 Oct 26;18(10):e3000881. doi: 10.1371/journal.pbio.3000881 (PMC7644108; doi:10.1371/journal.pbio.3000881)
Supplement: S2 Text — (PDF) [file pbio.3000881.s002.pdf]

Supporting Material and Methods

Tc-Rx antibody generation and verification

An antibody for the *Drosophila* Rx (Dm-Rx) protein was kindly gifted by Dr. Uwe Walldorf [1]. Its specificity was verified by absence of staining in Dm-Rx null mutant brains and by a similar expression pattern as *Dm-rx* RNA [1,2].

We tested cross-specificity of this antibody to the *Tribolium* Rx (Tc-Rx) protein. However, no staining was detected (data not shown). As the antigenic region of Dm-Rx used for antibody generation by [1] is absent or highly diverged in the *Tribolium* Rx protein (like in a number of other species, see S1 Fig) we used the Tc-Rx N-terminal region (amino acids 1-107), avoiding highly conserved homeobox and OAR domains to generate a suitable antibody. An N-terminal 321 bp gene sequence was amplified (primers including linker sequences: *Tc-rx-N_fw* and *Tc-rx-N_rev*, S2 Table) from wildtype cDNA and cloned into a Golden Gate vector containing a 6x His-Tag and a sequence encoding for a SUMO polypeptide (KNE001, pET SUMO-GoldenGate, S1 Vector) with a molar ratio of 1:5 of insert to vector (see below for source, modifications and cloning information).

For subsequent protein expression and purification, we essentially followed [3]. The vector was transformed into bacteria of the BL21-DE3 Rosetta strain. Bacteria expressed the peptide in TB (Terrific Broth) medium with the addition of 15 mM Glucose by 0.8 mM IPTG induction at an OD_{600} of 0.8 for four hours, were then harvested (5,000×g, 20 min, 4°C), resuspended in lysis buffer (50 mM Tris-HCl pH=7.5, 150 mM NaCl, 10 mM Imidazole), fractionated using a microfluidizer 110S (Microfluidics, MA, USA) and cell debris was removed by centrifugation (30,000×g, 30 min, 4°C). The peptide was subsequently purified by immobilized metal ion affinity chromatography using an ÄKTAprime plus and Nickel-charged affinity columns (both GE Healthcare Lifesciences, Chicago, USA). Main steps included affinity chromatography with a linear gradient of elution buffer (50 mM Tris-HCl pH=7.5, 150 mM NaCl, 400 mM Imidazole), cleavage of the His_6-SUMO tag with SUMO protease (1:50 molar ratio protease to peptide) with simultaneous dialysis (50 mM Tris-HCl pH=7.5, 150 mM NaCl) over night at 4°C, a second affinity chromatography to remove the His_6-SUMO tag and finally a size

exclusion chromatography with the Superdex 30 16/60 (GE Healthcare) and storage in 1X PBS. The purified protein fragment was used for polyclonal antibody generation and subsequent affinity purification of the antibody (Kaneka Eurogentec S.A., Belgium).

To exclude possible off-targets of the antibody and to validate whether the protein was correctly detected by the antibody [4], we performed a combination of *Tc-rx* in situ hybridisation (DIG-labelled full length probe, 0.4 µl in 30 µl hybridisation buffer) and Tc-Rx antibody staining in *Tribolium* embryos (S1 Fig)[5,6]. We found a high degree of overlap between the antibody staining and in situ hybridisation (S1 Fig). No additional staining in the embryo was observed, so that off-targets seem unlikely. To confirm specificity for the endogenous protein, we performed parental RNAi against *Tc-rx* (1.5 µg/µl) following standard procedures [5]. We then performed antibody stainings, including a control staining against Engrailed (to exclude differences in staining intensity) in knockdown and wildtype animals (S1 Fig). All steps from fixation to imaging were performed using a standardized protocol. Maximum intensity projections of 34 animals were grouped into three different Tc-Rx staining intensity groups. A blinded categorisation into wildtype and knockdown animals was performed and revealed that all knockdown animals belonged to middle or low strength categories confirming a reduction of Tc-Rx. Hence, the new antibody against Tc-Rx is highly specific for the provided antigen (affinity purification) and the endogenous protein (S1 Fig).

KNE001 cloning and map

The vector KNE001 (S1 Vector, pET SUMO-GoldenGate) was based on pET SUMOadapt (modified from the pET SUMO expression vector; [7–9]; material transfer agreement with Cornell University, U.S.A., ThermoFisher Scientific, MA, USA). This vector contained an adapter sequence with most importantly a *Bsa*I type IIS recognition site, allowing residue-free cloning of the CDS of interest in-frame with the *ATG::6xHis::SUMO* open reading frame. A second *Bsa*I site was then integrated by first amplifying a fragment additionally containing lac promoter, *CAT* gene and *ccdB* death cassette [10,11] with primers *GG_ccdB_F* and *GG_ccdB_R* (containing a *Xho*I-site) from pTALEN(NI)v2 (gift from Feng Zhang, Addgene Plasmid # 32189, [12]). Second, a *Not*I/*Xho*I digestion resulted in a 1.5 kb

*Not*I_*lacP-CAT_ccdB_Xho*I fragment, which was ligated into the *Not*I/*Xho*I linearized pET SUMOadapt. Third, the new pET SUMO-GoldenGate was transformed in ccdB Survival™ 2 T1R Competent Cells (ThermoFisher Scientific, MA, USA).

Hence, by adding GoldenGate linker sequences (S2 Table) that contain *Bsa*I cleavage sites (which do not equal the enzyme's recognition site) to the gene-specific forward and reverse primer, pET SUMO-GoldenGate and the CDS - in our case the N-terminal part of Tc-Rx - can be cut and ligated into a product lacking the original restriction sites. The ccdB cassette in the original KNE001 vector facilitates selection of clones with the gene fragment incorporated.

Generation of a *Drosophila* bicistronic Rx transgenic line

In order to generate a comprehensive picture of projections of all Dm-Rx-positive cells and to enable subsequent comparative development of Rx-positive cell groups, we generated a bicistronic line (S3 Fig) using the CRISPR/Cas9 technique [13,14]. We also screened available transgenic lines, i.e. two VT-GAL4 lines (https://stockcenter.vdrc.at) that include small fragments of the Dm-Rx regulatory region and hence only covered very small portions of Dm-Rx expression (data not shown).

We built a bicistronic construct as part of the CRISPR repair template, consisting of the C-terminal part of the *Dm-rx* gene, the CDS encoding for *EGFP* and a *P2A* peptide sequence [15,16]. The 22 amino acid long P2A peptide [15] is suggested to cause ribosomal skipping [17]. This sequence, if placed between two genes or CDS enables the transcription of one long mRNA of *Dm-rx-P2A-EGFP*, but the translation of two separate proteins. The *P2A* and *EGFP* sequences were inserted by using a guide RNA with the target sequence near the *Dm-rx* STOP codon (guide A, S3 Fig). This should result in a common expression of Dm-Rx and EGFP in the same cells, without disturbing the function of either gene through e.g., a fusion product, but with EGFP being in the cytoplasm and Dm-Rx retaining its nuclear localisation.

We included the fluorescent eye marker *3XP3-DsRed* [18]. Note that we avoided other eye or body markers, such as *mini-white* because of their size, which might reduce rates of homology-

directed repair further. In order to reduce the possible influence of the 3XP3 promotor on Dm-Rx or EGFP expression we inserted the eye marker in the downstream intergenic region, by using a gRNA targeting the intergenic region (guide B, S3 Fig). To facilitate homology-directed repair we included two flanking homology arms (S3 Fig, S2 Vector). As a result, our repair template consisted of seven fragments, which we assembled using a Gibson Assembly® kit (New England Biolabs, MA, USA), following the manufacturer's instructions:

1. Backbone: pJET 1.2/blunt (K1231, ThermoFisher Scientific, MA, USA), EcoRV linearized
2. left homology arm (F1 (Fragment 1): 1 kb of the C-terminus of *Dm-rx* (CG10052) excluding STOP codon
3. P2A peptide (F2): insect codon-optimized sequence (S2 Vector) from plasmid KNE020 (unpublished)
4. EGFP (F3): from plasmid gifted by the Wimmer department, University of Göttingen
5. 3' UTR and intergenic region from genomic DNA (F4): is the region between guide A and B3 (S3 Fig)
6. 3XP3-dsRED-SV40 (F5): eye marker, from plasmid gifted by the Wimmer department, University of Göttingen
7. Right homology arm (F6): 1 kb downstream of guide B3 cut site (three base pairs upstream of its PAM)

The target for guide A would thus be between F1 and F2, and the target for guide B between F5 and F6.

In order to identify single nucleotide polymorphisms in the target strain, integrate the right sequences for F1, F4, F6 and to identify suitable target sites and guide RNAs, we isolated genomic DNA (as described in [13]) from the *Act5C-Cas9, DNAlig4[169]* donor stock [19], and PCR amplified and sequenced the *Dm-rx* C-terminal region, 3' UTR and intergenic region (primers *DmRx_CDS_3'UTR_fw, DmRx_CDS_3'UTR_rev, DmRx_3'UTR_int-region_fw, DmRx_3'UTR_int-region_rev*, S2 Table). These regions were used to locate target sites (S3A^{iii} Fig) via the CRISPR Optimal Target Finder (http://targetfinder.flycrispr.neuro.brown.edu/). No off-targets were present

for all targets selected. Annealed oligonucleotides were cloned into a U6:3-*Bbs*I vector (based on pCFD3-dU6:3gRNA, Addgene #49410, [20], kindly provided by Hassan M.M. Ahmed (Wimmer department, University of Göttingen, unpublished)) via a GoldenGate reaction, following procedures in [13] but using *Bbs*I (New England Biolabs, MA, USA). Successful cloning was verified by sequencing the complete chimeric RNA scaffold (including trans-activating crRNA, [20]). guideRNAs were quality controlled by using a T7 Endonuclease I assay (see [13] for procedure). Injection procedures followed descriptions in [21].

Based on the T7 Endonuclease I assay, we selected one guide for the guide A target site, and three with overlapping target sites for the target of guide B (B1-3) (S3A^{iii} Fig).

Next, we designed a 1 kb long F1 (left homology arm) so that it ended before the STOP of *Dm-rx*, as Guide A caused a Cas9 cut only 8 bp downstream of the *Dm-rx* STOP. F4 was designed from the 3'UTR start to the cut site of guide B3 (note that guide B1 and B2 were near B3), with modifications of all PAMs in primers P7 and P8. F6 was 1 kb long, starting at the cut site of guide B3.

All fragments for the Gibson Assembly® were amplified using the primers P1 to P12 (S2 Table), containing appropriate overlaps to the neighbouring fragment. F1, 4 and 6 were amplified from the previously isolated genomic DNA of *Act5C-Cas9, DNAlig4 [169]* line. We then used three assembly reactions (roman numerals in primers P1 to P12). The first assembled F1 to F3, the second F4 to F6, and the third assembled the products of the first two reactions.

The four plasmids containing each guide and MF01 were precipitated [13,21] to ensure DNA purity and increase viability of embryos after injection. We then made three injection mixes, each containing one of the guides B1 to B3 (250 ng/µl), guide A (250 ng/µl) and MF01 (400 ng/µl), diluted in 1x injection buffer [21]. Subsequent injections followed descriptions in [21].

We injected 1203 embryos of which 424 G_0 adults survived. We crossed them singly to three w^{1118} virgins of the opposite sex of which 224 G_1 crossings gave rise to offspring. We then screened them under a fluorescence stereo microscope (Leica M205 FA, Leica, Wetzlar, Germany) for the presence of the 3XP3-DsRed eye marker and identified 27 positive independent lines. Note, however, that we observed heritable variability in strength and location of DsRed inside the *Drosophila* eye.

We thus took four of the 27 positive stocks, with varying degree of eye marker strength and screened wandering third instar larval brains for any detectable differences in the presence of a GFP fluorescence signal resembling known Dm-Rx antibody staining [1]. All four stocks did not vary in GFP expression and showed equal similarity to a Dm-Rx antibody staining. To verify this tendency, we performed immunostainings in offspring embryos of these four lines and detected GFP and Dm-Rx signal through a GFP antibody staining. Embryos of all four stocks showed near 100 % overlap to Dm-Rx and a cytoplasmic signal. Finally, to verify that insertion was performed as planned (S3A^{ii} Fig), we isolated genomic DNA from one whole adult male of each of the four stocks using the Zymo Research *Quick*-DNA Miniprep Plus kit (Zimo Research, Irvine, CA, USA) following the manufacturer's Solid Tissues Protocol. We then amplified DNA fragments containing the whole region by nested PCR (primers *DmRx_trans-ver_fw, DmRx_trans-ver_rev, DmRx_trans-ver_nested_fw, DmRx_trans-ver_nested_rev*, S2 Table). We sequenced the regions surrounding the cut sites with primers *DmRx_trans_seq_Ct_fw* and *DmRx_trans_seq_iRe_rev* (S2 Table). All four stocks showed correct sequencing at guide A cut sites, but only the line used in this study showed completely correct sequences, thus allowing us to perform suitable experiments and closer characterisation (S3C-F Fig).

To verify that EGFP is indeed localised in the cytoplasm, we performed immunostainings for Dm-Rx and GFP in embryos. With higher magnification we were able to see a substantial amount of GFP in the cytoplasm surrounding the nuclei marked by Dm-Rx (S3C Fig) and DAPI (not shown). We also wanted to know whether expression from the transgenic Dm-Rx locus was qualitatively different from the endogenous expression, so to ensure that we investigated Dm-Rx expression similar to a wildtype situation. For this we performed immunostainings against Dm-Rx in the adult *Drosophila* brain with identical settings and imaged them identically (S3D Fig). We were not able to detect any absence of domains (S3D Fig). Differences between the wildtype strain w^{1118} and our transgenic line were – if present – as large as differences between individual brains of the same genetic background.

We then tested whether the expression of Dm-Rx and EGFP in the same cells is maintained in the adult brain (S3E-F Fig). Indeed, by qualitative assessment we were able to see an approximately 100 % coexpression, with prominent projections marked as well (Fig 3).

We thus concluded that the Rx-GFP bicistronic line was suited for our use.

Characterisation and validation of the *Tribolium* Rx-GFP enhancer trap

We identified a suitable *Tribolium* transgenic line in the GEKU base website (# E01101, http://www.geku-base.uni-goettingen.de/; [22]). Insertion had been mapped to the upstream region of *Tc-rx* ([22]; S2A Fig). To identify to which degree Tc-Rx expressing cells also express GFP we performed co-stainings in adult brains. We found that n-ventral Tc-Rx-positive cells were not marked by the line at all while n-dorsal domains were only partially marked (S4 Fig). However, by manually checking each GFP expressing cell, we found that all cells expressing GFP in the region surrounding the protocerebral bridge, also expressed Tc-Rx (S2B-D/F Fig). Hence, there were no cells that were marked false-positively. Interestingly, there were more GFP expressing cells showing overlap to Tc-Rx expression in all other stages of development (S2E/F Fig). Manual quantification was performed by first limiting image data to the region of interest in all three dimensions (S2F^{i} Fig). Then, background was removed by modifying brightness and contrast. Then, we determined how many slices approximately covered one cell and made small Z projections. Using the Cell Counter plugin of ImageJ, we counted the number of cells in the projection. We exported the markers and then we overlapped these markers with the next progressive Z projection to avoid counting any cell twice. Cells were counted, means and standard deviations calculated for N=3 brains.

To ensure that Tc-Rx is expressed similar to the wildtype situation, we performed identical immunostainings against Tc-Rx and imaging with identical settings in the transgenic line and wildtype v^{w} background (S2C Fig)[23]. We found that differences between conditions were no larger than the differences observed between individuals of the same condition. We thus concluded that the Rx-GFP enhancer trap was suitable for further experiments.

Generation of homozygous stocks of Rx-GFP transgenic lines

A homozygous stock of the *Tribolium* Rx-GFP enhancer trap was generated by genotyping adult wing tissue, as described in [13,24], with primers *GEKU-Rx-GFP_wt_fw*, *GEKU-Rx-GFP_wt_rev*, *GEKU-Rx-GFP_trans_rev* (S2 Table).

A homozygous stock of the *Drosophila* Rx-GFP bicistronic line was generated by crossing the male offspring (G_2) of the G_1 cross to female virgins of a w^-*; wg*$^{Gla-1}$*/CyO* 2nd chromosome balancer (a kind gift by the Wimmer department, University of Göttingen). CyO/w^- positive animals (G3) were selected and crossed to each other, to create homozygous positive animals (G4) for the transformation marker (*3XP3-dsRed-SV40*).

Both transgenic lines were viable in the homozygous background.

R45F08-GAL4 crosses

To reveal the overlap of secondary cells of the DM1-3 and 6 lineages marked by the R45F08-GAL4 line [25,26] with Dm-Rx expressing cells we performed two crosses and subsequent immunostainings (S5 Fig).

First, we crossed the R45F08-GAL4 line with a UAS-mcD8::GFP line and investigated offspring third instar larvae to visualize the characterized cells and subsequently stained with anti-GFP and anti-Rx antibodies (S5A-B Fig).

Second, to visualize an overlap of Dm-Rx expressing cells and R45F08 labelled cells, we first crossed the *Drosophila* Rx-GFP bicistronic line each separately with R45F08-GAL4 and UAS-mcD8::RFP. The respective offspring was then crossed to each other. We then dissected 15 brains of third instar larvae, stained them with anti-RFP and anti-GFP, screened for the presence of RFP and GFP label and imaged double-positive brains (S5C Fig).

Staging of *Tribolium* and *Drosophila* animals

Table S6 displays all stages and their description included in our study. Particularly pupal staging and the late larval stages were determined using time (which allowed us to calculate relative times of pupation) and morphology as criteria to confirm the timed staging.

Drosophila embryonic stages were determined using the staging of [27] and pupal stages using staging in [28] (S6 Table displays the most important pupal selection criteria). Eye colouring and morphology were not included in staging due to the w^{1118} background. Information on the length of embryonic development used in Fig 10 was derived from [27]. Length of larval development and pupation was measured for our Rx-GFP bicistronic line specifically.

Tribolium embryonic stages were determined using the staging of [29] and for late embryonic stages using staging of Scholten and Klingler (unpublished). *Tribolium* pupal and late larval staging was aided by [30] and Dippel (unpublished). Information on length of embryonic development used in Fig 10 was derived from [29] and Scholten and Klingler (unpublished). Total developmental time was taken from [31]. Larval and pupal developmental length was measured for our Rx-GFP enhancer trap specifically. To that end, we first determined the duration of pupation in the used Rx-specific transgenic lines. Pupation in the *Tribolium* Rx-GFP line took approximately 140 h, while pupation in the *Drosophila* Rx-GFP line took approximately 100 h. A developmental progress of 5 % equals 7 h in *Tribolium*, and 5 h in *Drosophila*.

Specimen fixation and immunohistochemistry

Methanol fixation of *Drosophila* embryos was performed following standard protocols [32]. Fixation of *Tribolium* embryos was based on [33] with following modifications: Fixation was performed with 2 ml of fixation buffer PEMS (0.1 M PIPES, 2 mM MgCl, 5 mM EGTA, pH = 6.9); we added 180 µl of 37 % formaldehyde (F 1635, Merck, Darmstadt, Germany) and fixed embryos between 25 and 32 min; devitellinisation was first conducted with a 0.9 µm canula, for older stages (> 40 h) we followed with a 0.8 µm canula.

Immunohistochemistry of embryos was based on procedures in [6], with the addition of preceding washes in a descending methanol series (75, 50 and 25 % Methanol with PBS-T 0.1 %), followed by two rinse steps and three 10 min washes.

For all stainings normal goat serum was used as blocking solution (NGS, G9023, Merck, Darmstadt Germany, see S3 Table). Fixative for all other stages except for embryos was 4 % PFA (wt/vol, paraformaldehyde, (e.g. P6148, Merck, Darmstadt, Germany) in PBS, 130 mM NaCl, 7 mM Na_2HPO_4, 3 mM KH_2PO_4, [34]). Washing buffer for all stages except embryos was phosphate buffer (PB, see [35] for recipe). Brains were dissected using Dumont No. 5 forceps in ice-cold PB. All steps were performed in 180 µl volume in 9-well PYREX™ Spot Plates (ThermoFisher Scientific, MA, USA) on an orbital shaker. Protocols were adapted from [34,35].

Image acquisition, processing and 3D reconstruction

If not otherwise specified, imaging was performed at a Leica SP8 confocal microscope (Wetzlar, Germany). Objectives used were either a Leica apochromat 20x (NA = 0.75) or a 63x HC PL APO CS2 (NA = 1.30) glycerol-immersion objective. DAPI was excited by a Diode laser (405 nm), Alexafluor 488 (ThermoFisher Scientific, MA, USA) by an Argon laser (488 nm), Alexafluor 555 by a DPSS laser (561 nm) and Alexafluor 647 by a HeNe laser (633 nm). Detection was performed with Hybrid detectors and photomultipliers, at an 8-bit depth. Averaging was depending on which staining was performed, set on line or frame averaging of 4. Step size was set to system optimized values defined by the LASX software. Image size was set between 1,024 x 1,024 and 2,048 x 2,048 pixels. Images were processed, adjusted for brightness and contrast, cropped, merged and rotated using the Fiji software [36]. Maximum intensity projections and smooth manifold extractions (SMEs; [37]) to retain 3D spatial relationships were calculated using Fiji as well [36].

3D reconstructions were performed in Amira 5.4.1 (Visage Imaging, Fürth, Germany). We created Labelfield data with the same pixel and voxel size resolution as the original data set. We then used the Segmentation Editor to identify and create material for each tract and central complex neuropils by employing the Wand tool. Subsequent marking was modified for visual ease using the grow, fill

holes and smooth functions of the Segmentation Editor. Subsequently we created 3D surfaces with the Surface Generator.

In some cases, projections were too thin to be recapitulated in the 3D surface. For this, where we logically inferred a connection of axons that was only faintly marked by the original file, we used the Brush tool.

We only reconstructed the axon connections to certain cell bodies where we were sure that they are directly connected. This excluded a few cell bodies from the analysis, particularly in the *Drosophila* adult brain.

References

1. Davis RJ, Tavsanli BC, Dittrich C, Walldorf U, Mardon G. Drosophila retinal homeobox (drx) is not required for establishment of the visual system, but is required for brain and clypeus development. Dev Biol. 2003;259: 272–287. doi:10.1016/S0012-1606(03)00201-X

2. Eggert T, Hauck B, Hildebrandt N, Gehring WJ, Walldorf U. Isolation of a Drosophila homolog of the vertebrate homeobox gene Rx and its possible role in brain and eye development. PNAS. 1998;95: 2343–2348.

3. Monecke T, Buschmann J, Neumann P, Wahle E, Ficner R. Crystal Structures of the Novel Cytosolic 5′-Nucleotidase IIIB Explain Its Preference for m7GMP. PLOS ONE. 2014;9: e90915. doi:10.1371/journal.pone.0090915

4. Uhlen M, Bandrowski A, Carr S, Edwards A, Ellenberg J, Lundberg E, et al. A proposal for validation of antibodies. Nat Methods. 2016;13: 823–827. doi:10.1038/nmeth.3995

5. Posnien N, Schinko J, Grossmann D, Shippy TD, Konopova B, Bucher G. RNAi in the Red Flour Beetle (Tribolium). Cold Spring Harb Protoc. 2009;2009: pdb.prot5256. doi:10.1101/pdb.prot5256

6. Buescher M, Oberhofer G, Garcia-Perez NC, Bucher G. A Protocol for Double Fluorescent In Situ Hybridization and Immunohistochemistry for the Study of Embryonic Brain Development in Tribolium castaneum. In: Sprecher SG, editor. Brain Development. New York, NY: Springer New York; 2020. pp. 219–232. doi:10.1007/978-1-4939-9732-9_12

7. Bosse-Doenecke E, Weininger U, Gopalswamy M, Balbach J, Knudsen SM, Rudolph R. High yield production of recombinant native and modified peptides exemplified by ligands for G-protein coupled receptors. Protein Expr Purif. 2008;58: 114–121. doi:10.1016/j.pep.2007.10.012

8. Hanington PC, Barreda DR, Belosevic M. A Novel Hematopoietic Granulin Induces Proliferation of Goldfish (Carassius auratus L.) Macrophages. J Biol Chem. 2006;281: 9963–9970. doi:10.1074/jbc.M600631200

9. Mossessova E, Lima CD. Ulp1-SUMO Crystal Structure and Genetic Analysis Reveal Conserved Interactions and a Regulatory Element Essential for Cell Growth in Yeast. Molecular Cell. 2000;5: 865–876. doi:10.1016/S1097-2765(00)80326-3

10. Bernard P, Gabarit P, Bahassi EM, Couturier M. Positive-selection vectors using the F plasmid ccdB killer gene. Gene. 1994;148: 71–74. doi:10.1016/0378-1119(94)90235-6

11. Engler C, Kandzia R, Marillonnet S. A One Pot, One Step, Precision Cloning Method with High Throughput Capability. PLOS ONE. 2008;3: e3647. doi:10.1371/journal.pone.0003647

12. Sanjana NE, Cong L, Zhou Y, Cunniff MM, Feng G, Zhang F. A transcription activator-like effector toolbox for genome engineering. Nature Protocols. 2012;7: 171–192. doi:10.1038/nprot.2011.431

13. Farnworth MS, Eckermann KN, Ahmed HMM, Mühlen DS, He B, Bucher G. The Red Flour Beetle as Model for Comparative Neural Development: Genome Editing to Mark Neural Cells in Tribolium Brain Development. In: Sprecher SG, editor. Brain Development. New York, NY: Springer New York; 2020. pp. 191–217. doi:10.1007/978-1-4939-9732-9_11

14. Gratz SJ, Wildonger J, Harrison MM, O'Connor-Giles KM. CRISPR/Cas9-mediated genome engineering and the promise of designer flies on demand. Fly. 2013;7: 249–255. doi:10.4161/fly.26566

15. Kim JH, Lee S-R, Li L-H, Park H-J, Park J-H, Lee KY, et al. High Cleavage Efficiency of a 2A Peptide Derived from Porcine Teschovirus-1 in Human Cell Lines, Zebrafish and Mice. PLOS ONE. 2011;6: e18556. doi:10.1371/journal.pone.0018556

16. Szymczak-Workman AL, Vignali KM, Vignali DAA. Design and Construction of 2A Peptide-Linked Multicistronic Vectors. Cold Spring Harbor Protocols. 2012;2012: pdb.ip067876-pdb.ip067876. doi:10.1101/pdb.ip067876

17. Donnelly MLL, Luke G, Mehrotra A, Li X, Hughes LE, Gani D, et al. Analysis of the aphthovirus 2A/2B polyprotein 'cleavage' mechanism indicates not a proteolytic reaction, but a novel translational effect: a putative ribosomal 'skip.' J Gen Virol. 2001;82: 1013–1025. doi:10.1099/0022-1317-82-5-1013

18. Berghammer AJ, Klingler M, Wimmer EA. A universal marker for transgenic insects. Nature. 1999;402: 370. doi:10.1038/46463

19. Zhang X, Koolhaas WH, Schnorrer F. A Versatile Two-Step CRISPR- and RMCE-Based Strategy for Efficient Genome Engineering in Drosophila. G3: Genes, Genomes, Genetics. 2014;4: 2409–2418. doi:10.1534/g3.114.013979

20. Port F, Chen H-M, Lee T, Bullock SL. Optimized CRISPR/Cas tools for efficient germline and somatic genome engineering in Drosophila. PPNAS. 2014;111: E2967–E2976. doi:10.1073/pnas.1405500111

21. Eckermann KN, Ahmed HMM, KaramiNejadRanjbar M, Dippel S, Ogaugwu CE, Kitzmann P, et al. Hyperactive piggyBac transposase improves transformation efficiency in diverse insect species. Insect Biochem Mol Biol. 2018;98: 16–24. doi:10.1016/j.ibmb.2018.04.001

22. Trauner J, Schinko J, Lorenzen MD, Shippy TD, Wimmer EA, Beeman RW, et al. Large-scale insertional mutagenesis of a coleopteran stored grain pest, the red flour beetle Tribolium

castaneum, identifies embryonic lethal mutations and enhancer traps. BMC Biol. 2009;7: 73. doi:10.1186/1741-7007-7-73

23. Lorenzen MD, Brown SJ, Denell RE, Beeman RW. Cloning and characterization of the Tribolium castaneum eye-color genes encoding tryptophan oxygenase and kynurenine 3-monooxygenase. Genetics. 2002;160: 225–234.

24. Strobl F, Ross JA, Stelzer EHK. Non-lethal genotyping of Tribolium castaneum adults using genomic DNA extracted from wing tissue. Papadopoulos NT, editor. PLOS ONE. 2017;12: e0182564. doi:10.1371/journal.pone.0182564

25. Jenett A, Rubin GM, Ngo T-TB, Shepherd D, Murphy C, Dionne H, et al. A GAL4-Driver Line Resource for Drosophila Neurobiology. Cell Reports. 2012;2: 991–1001. doi:10.1016/j.celrep.2012.09.011

26. Riebli N, Viktorin G, Reichert H. Early-born neurons in type II neuroblast lineages establish a larval primordium and integrate into adult circuitry during central complex development in Drosophila. Neural Dev. 2013;8: 6. doi:10.1186/1749-8104-8-6

27. Campos-Ortega JA, Hartenstein V. The Embryonic Development of Drosophila melanogaster. New York: Springer-Verlag; 1985.

28. Bainbridge SP, Bownes M. Staging the metamorphosis of Drosophila melanogaster. J Embryol Exp Morphol. 1981;66: 57–80.

29. Biffar L, Stollewerk A. Conservation and evolutionary modifications of neuroblast expression patterns in insects. Dev Biol. 2014;388: 103–116. doi:10.1016/j.ydbio.2014.01.028

30. Ho FK. Optic Organs of Tribolium confusum and T. castaneum and Their Usefulness in Age Determination (Coleoptera: Tenebrionidae). Ann Entomol Soc Am. 1961;54: 921–925. doi:10.1093/aesa/54.6.921

31. Sokoloff A. The Biology of Tribolium. Oxford: Clarendon Press; 1974.

32. Rothwell WF, Sullivan W. Fixation of Drosophila Embryos. Cold Spring Harb Protoc. 2007;2007: pdb.prot4827. doi:10.1101/pdb.prot4827

33. Schinko J, Posnien N, Kittelmann S, Koniszewski N, Bucher G. Single and Double Whole-Mount In Situ Hybridization in Red Flour Beetle (Tribolium) Embryos. Cold Spring Harb Protoc. 2009;2009: pdb.prot5258. doi:10.1101/pdb.prot5258

34. Riemensperger T, Isabel G, Coulom H, Neuser K, Seugnet L, Kume K, et al. Behavioral consequences of dopamine deficiency in the Drosophila central nervous system. PNAS. 2011;108: 834–839. doi:10.1073/pnas.1010930108

35. Ostrovsky A, Cachero S, Jefferis G. Clonal Analysis of Olfaction in Drosophila: Immunochemistry and Imaging of Fly Brains. Cold Spring Harb Protoc. 2013;2013: pdb.prot071720. doi:10.1101/pdb.prot071720

36. Schindelin J, Arganda-Carreras I, Frise E, Kaynig V, Longair M, Pietzsch T, et al. Fiji: an open-source platform for biological-image analysis. Nat Methods. 2012;9: 676–682. doi:10.1038/nmeth.2019

37. Shihavuddin A, Basu S, Rexhepaj E, Delestro F, Menezes N, Sigoillot SM, et al. Smooth 2D manifold extraction from 3D image stack. Nat Commun. 2017;8: 15554. doi:10.1038/ncomms15554
