# Supplementary figures and images for: Sequence heterochrony led to a gain of functionality in an immature stage of the central complex: A fly–beetle insight
Source: PLoS Biol. 2020 Oct 26;18(10):e3000881. doi: 10.1371/journal.pbio.3000881 (PMC7644108; doi:10.1371/journal.pbio.3000881)

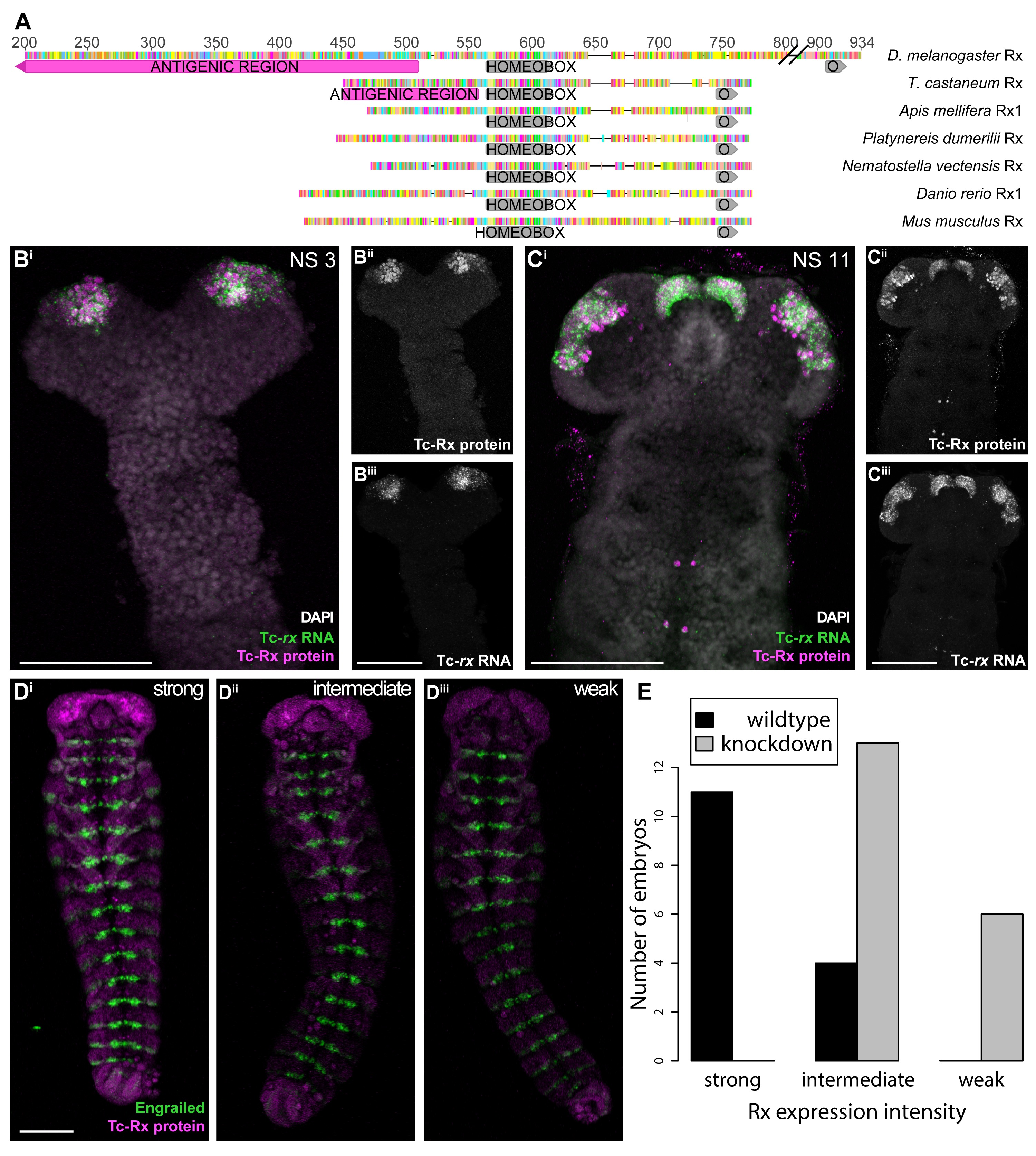

Supplement: S1 Fig — (A) Alignment (Geneious 11.1.5, Geneious Alignment) of Rx proteins of Drosophila and Tribolium as well as representative species. The conserved homeobox and OAR (O) domains (gray) are present in all proteins. Antigenic regions for the Dm-Rx [51,53] and the Tc-Rx antibody are displayed in magenta. The Dm-Rx protein was shortened for better display (amino acids 1 to 200 and most between 800 and 900 are not displayed). Notice that the D. melanogaster antigenic region appears to be absent in T. castaneum and all other species. (B-C) Tc-Rx protein and Tc-rx RNA expression in Tribolium embryos of neurogenesis stages 3 and 11 [37] were depicted (Zeiss LSM510, 40× immersion objective) as maximum intensity projections (DAPI for structure as average projection). Anterior is to the top. Animals were mounted dorsal up. The signal detected in the antibody staining against Tc-Rx protein (magenta) overlapped to a high degree with the signal detected in the in situ hybridization (green). Note that although the protein of Tc-Rx was located in the nucleus, Tc-rx RNA was also in the cytoplasm of the cell soma, which resulted in a different cellular localization. (D) To validate the specificity of the Tc-Rx antibody, we performed a RNAi-mediated Tc-rx knockdown. Indeed, Tc-Rx expression was reduced in knockdown embryos. Depicted are 3 categories of Tc-Rx expression (i.e., Tc-Rx antibody staining intensity, magenta, as maximum intensity projections) after knockdown (strong, equaling wildtype, in Di, intermediate in Dii, weak in Diii). To accommodate for differences in intensity of staining, a co-staining against Invected/Engrailed with the respective antibody was performed. (E) A total of 34 RNAi embryos were categorized into the 3 expression intensity groups in a blinded experiment. Wild-type animals showed a high level of expression and were mostly grouped in category “strong” with some in category “intermediate.” No knockdown animals were grouped into the “strong” category, most in “ [file pbio.3000881.s005.tif]

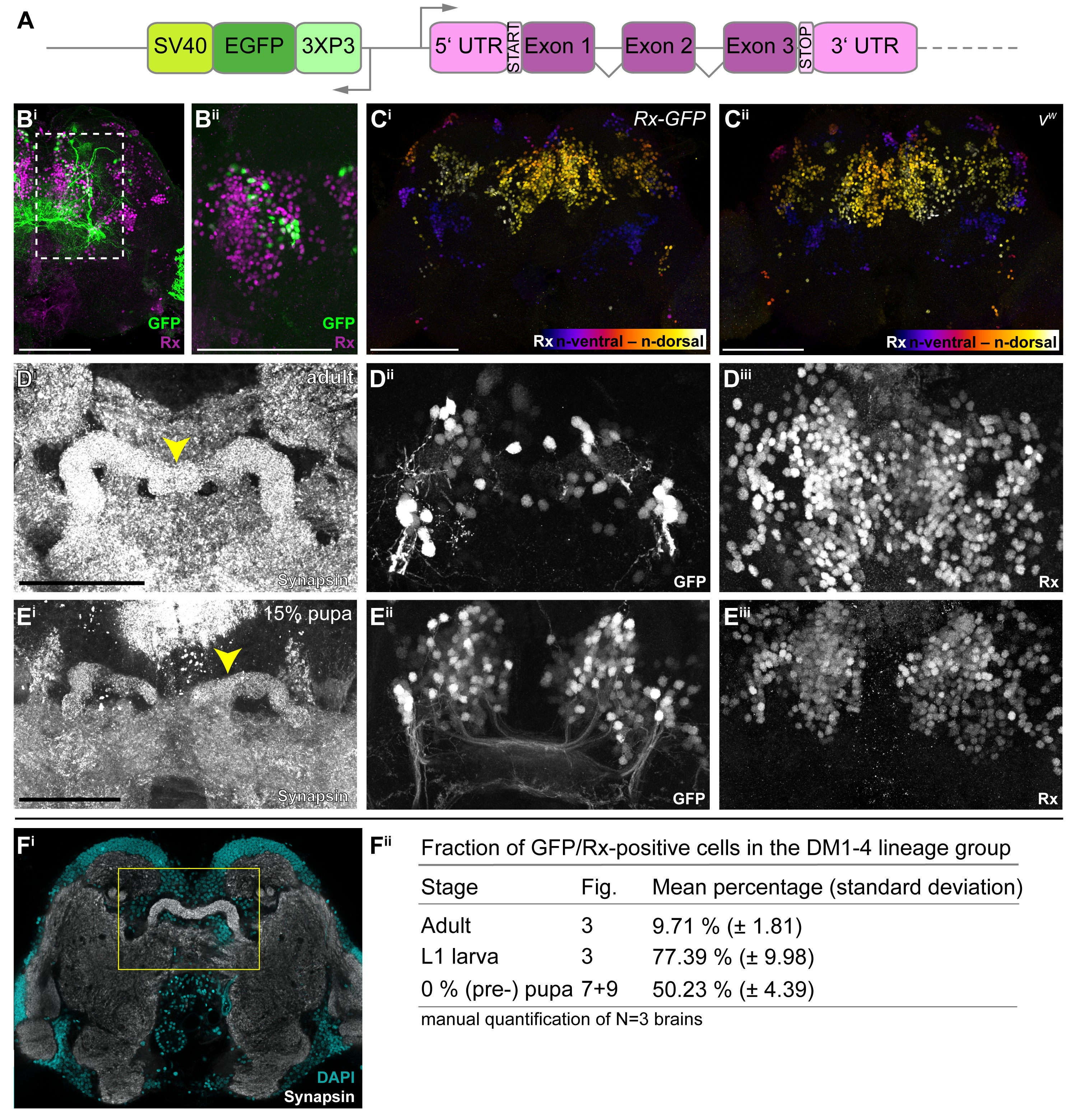

Supplement: S2 Fig — (A) The Tribolium rx-EGFP enhancer trap was taken from the GEKU screen collection [47] where enhancer traps were generated by piggyBac-mediated transposition. A 3XP3-EGFP-SV40 cassette randomly inserted upstream of the Tc-rx gene in opposite direction (insertion site mapped by [47]). (B) Maximum intensity projections of immunostainings against GFP and Tc-Rx in adult brains of the Tc-rx-EGFP line. The line only marked a subset of Tc-Rx expressing cells. This also applies to the n-dorsal region (Bii). However, all EGFP-expressing cells also expressed Tc-Rx. Coexpression was verified manually. (C) The introduction of the enhancer trap cassette did not visually influence Tc-Rx expression, as domains were highly similar between transgenic Rx-GFP (Bi) and wild-type vermillion-white (vw, Bii, [90]) animals, as visualized by color-coded maximum intensity projections. Observed qualitative differences in Tc-Rx expression in the transgenic or wildtype condition (N = 3 each) were approximately as large as the differences between the genetic backgrounds. (D) A crop of a maximum intensity projection of cells surrounding the adult protocerebral bridge (yellow arrowhead, Di) shows the coexpression of GFP (Dii) and Tc-Rx (Diii) in a subset of cells that were subsequently used in this study. (E) An analogous analysis in young pupal brains of cells surrounding the protocerebral bridge (Ei) revealed more EGFP-expressing cells (Eii) with overlap to Tc-Rx cells (Eiii) than in the adult (D). (F) Quantification of Rx/GFP double-positive cells in the region of DM1-4 lineages surrounding the protocerebral bridge (yellow rectangle, Fi) revealed that at different developmental stages, the fraction of double-positive cells is different, ranging from approximately 10% to 75%. Scale bars in B and C represent 100 μm, and in D and E, scale bars represent 50 μm. EGFP, enhanced green fluorescent protein; GFP, green fluorescent protein; Rx, retinal homeobox protein. (TIF) [file pbio.3000881.s006.tif]

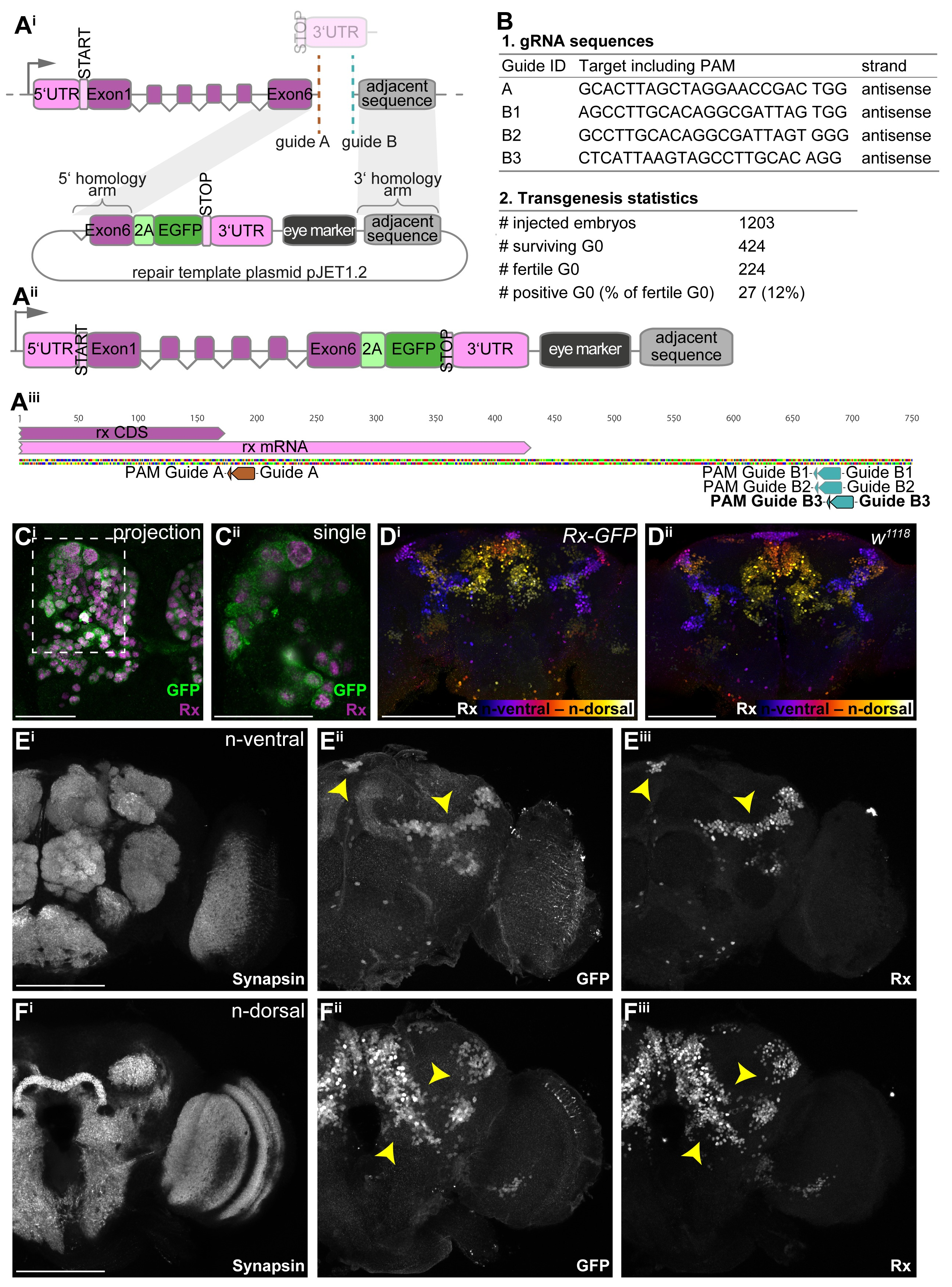

Supplement: S3 Fig — (Ai) Strategy of building a Dm-rx-EGFP line (modified from [39]). Two gRNAs next to the endogenous STOP codon (guide A, brown dashed line) and downstream of the Dm-rx 3’UTR (guide B, blue dashed line) were used. The DNA repair template included a sequence encoding for a P2A self-cleaving peptide, EGFP, the endogenous region between guide A and B (Dm-rx 3’UTR and a fraction of intergenic region), and the 3xP3-DsRed-SV40 eye marker, as well as 1-kb homology arms flanking the insertion sites. (Aii) The edited transgenic locus comprises a common open reading frame of both Dm-rx and EFGP with a STOP after EGFP. (Aiii) Four gRNAs were used in different combinations to generate similar transgenic lines. The gRNAs used for the transgenic line used in this study are marked in bold (guide A and B3). (B) Overview of gRNA sequences and transgenesis statistics upon injection [88] for the Drosophila Rx-GFP transgenic line. (Ci) Immunostaining of anti-Dm-Rx (magenta) and anti-GFP (green) in the Dm-rx-EGFP line showed that all visible cells that expressed Dm-Rx also expressed GFP, shown in a smooth manifold extension (SME) projection [66] of a brain hemisphere of a S16 embryo. The region marked with a dotted line in Ci is shown in (Cii) as a single slice. Here, the different cellular localizations are visible. Dm-Rx retained its nuclear localization, while GFP located to the cytoplasm, demonstrating functionality of the P2A peptide. (D) The transgenic line had normal Dm-Rx expression, shown by anti-Dm-Rx immunostaining and depth color-coded maximum intensity projection in the Rx-GFP line (Di) and the origin wildtype strain w1118 (Dii). Observed qualitative differences in Dm-Rx expression in the transgenic or wildtype condition (N = 3 each) were approximately as large as the differences between the genetic backgrounds. (E-F) Dm-Rx and EGFP expression matched in adult brains (see yellow arrowheads for exemplary double-positive areas). Maximum intensity projections of synapsin immunos [file pbio.3000881.s007.tif]

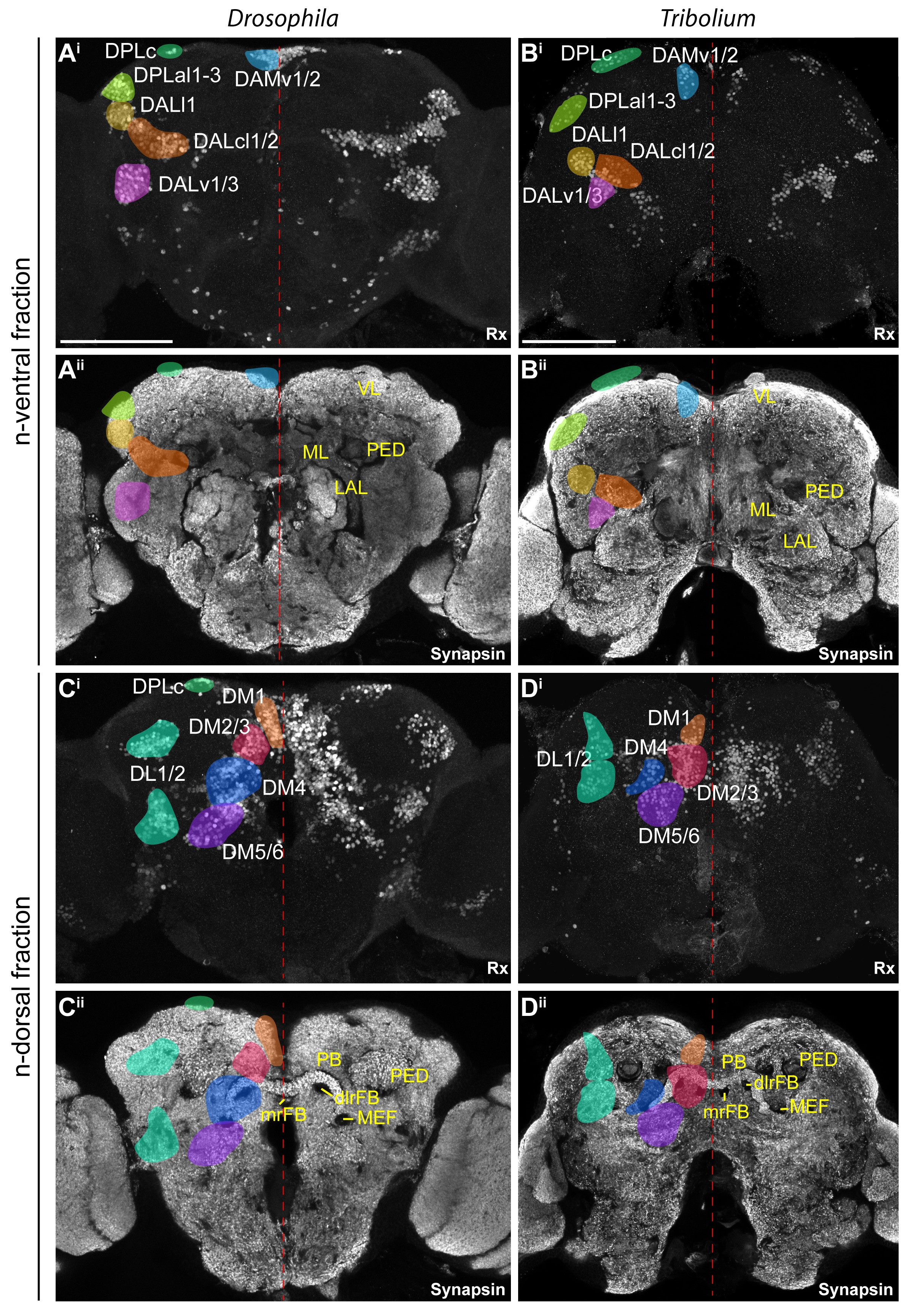

Supplement: S4 Fig — We mapped the labeled Rx-positive cells to previously described lineages of the Drosophila brain using locations relative to other brain structures and their projection pattern as criterion ([63, 64]; www.mcdb.ucla.edu/Research/Hartenstein/dbla/index.html and references therein). We tentatively named Tribolium cell clusters by using similar locations and projections as compared with the Drosophila atlas, used as guide. A list of all lineages with names and descriptions can be found in S1 Table. Hemispheres are separated by a red dotted line for orientation. Because of the cell body rind expression of Rx, domains and proposed lineages could be separated into 2 fractions, n-ventral and n-dorsal, corresponding to each half of the insects’ brains. For each species, 1 image stack was used and separated into 2 fractions. Rx expression is displayed by a maximum intensity projection of a substack of an anti-Rx immunostaining (i). Basic anatomical structure of the insects’ brains is displayed by a SME projection [66] of a synapsin immunostaining (ii). On this projection, in the left hemisphere, the locations of the proposed lineages are shown color-coded, whereas on the right hemispheres, basic anatomical structures are annotated that assist understanding differences in domain position between the species (yellow). dlrFB, dorso-lateral root of the FB; LAL, lateral accessory lobes; MEF, medial equatorial fascicle; ML, medial lobe; mrFB, medial root of the fan-shaped body; PB, protocerebral bridge; PED, peduncle; VL, vertical lobe. Scale bars represent 100 μm. (TIF) [file pbio.3000881.s008.tif]

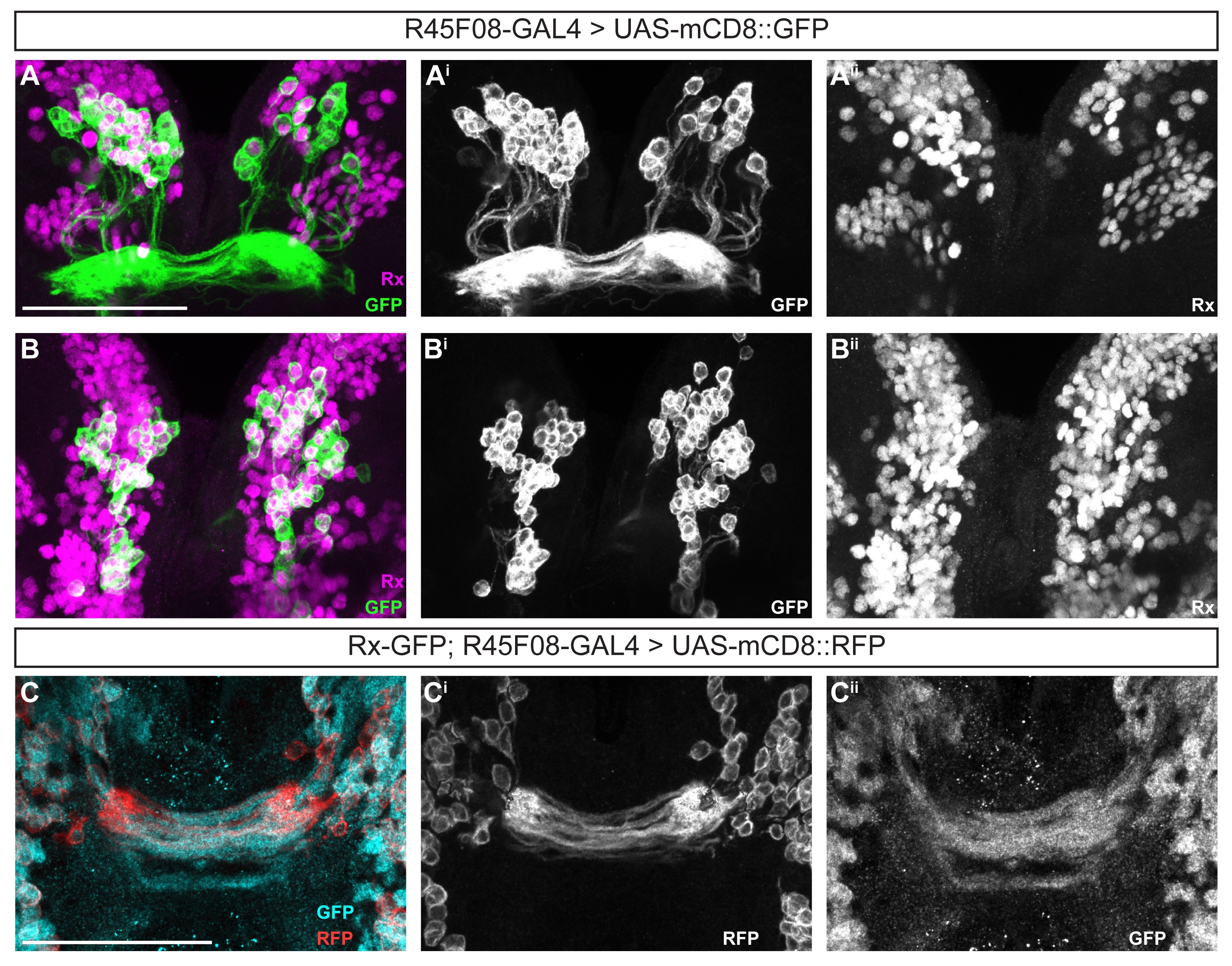

Supplement: S5 Fig — Displayed is a co-localization of Dm-Rx-positive neural cells and cells under the control of R45F08-GAL4 [13,91] shown in brains of Drosophila wandering third instar larvae. (A-B) Antibody staining in a cross of the R45F08-GAL4 line and UAS-mCD8::GFP was performed against Dm-Rx (depicted in magenta) and GFP (green) to reveal the coexpression of cell bodies of lineages DM1-3/6, marked through the R45F08-GAL4 line, and Dm-Rx. Approximately 90% of the R45F08-GAL4 GFP-positive cells were Dm-Rx-positive as well (A-Aii first half, B-Bii second half of the stack). (C) Antibody staining in animals (N = 2) of the respective cross from subcrosses of the Rx-GFP line each with R45F08-GAL4 line and the UAS-mCD8::RFP (SMEs, see [66]). This resulted in a coexpression of GFP in a Dm-Rx expression pattern and RFP under control of R45F08-GAL4. Antibody staining against GFP (cyan) and RFP (red) revealed coexpression of both fluorescent proteins in midline crossing projections. Although RFP is membrane-bound and GFP cytoplasmic, there were several fascicles showing coexpression of RFP and GFP. This corroborated the high degree of overlap of Dm-Rx and DM1-3/6 lineage offspring shown in panels A and B. Scale bars represent 50 μm. GFP, green fluorescent protein; Rx, retinal homeobox protein; SME, smooth manifold extraction. (TIF) [file pbio.3000881.s009.tif]
