# Supplementary material for: Sequence heterochrony led to a gain of functionality in an immature stage of the central complex: A fly–beetle insight
Source: PLoS Biol. 2020 Oct 26;18(10):e3000881. doi: 10.1371/journal.pbio.3000881 (PMC7644108; doi:10.1371/journal.pbio.3000881)
Supplement: Attachment — Submitted filename: Farnworth-et-al_Revision_Comments_final.docx.pdf [file pbio.3000881.s017.pdf]

Reviewers' comments

Rev. 1:

In this study, we investigated holometabolous insects, where larvae differ dramatically from the adult in both behavior and morphology. As consequence, the central complex, mainly responsible for spatial orientation, is conserved between species at the adult stage, but differs between larvae and adults as well as between larvae of different taxa.

1. We used genome editing and established transgenic lines to visualize cells expressing the conserved transcription factor retinal homeobox, thereby marking homologous genetic neural lineages in both the fly Drosophila melanogaster and the beetle Tribolium castaneum.

2. We compared the development of homologous neural cells between taxa from embryo to the adult, finding heterochronic changes including shifts of developmental events between embryonic and pupal stages.

3. We show sequence heterochrony in brain development, where developmental steps changed their position within the ontogenetic progression.

4. We show that through this sequence heterochrony, an immature developmental stage of the central complex gains functionality in Tribolium larvae.

Major comments

1. I liked the goal of this study, and appreciated the detailed analysis of both Drosophila and Tribolium developmental neuroanatomy. This type of study is foundational for further experimental analysis. The timelines in Figure 7 are beautiful and helpful.

Thank you – we are glad you enjoyed it.

2. Unfortunately, the anatomical images were not convincing. In Figure 3, the PB looks ok but the CBU and NO would not be well defined without the the surrounding lines. The CBL is even less well defined. Perhaps in a supplement or the reviewer repsonse there can be some un-outlined images to show the borders of each neuropil more clearly? I have similar issues (can't see the anatomical structures clearly) in figures 4 and 5 as well.

We are convinced that the given outlines are well supported by our data:

1) We identified them based on differences in Synapsin staining (this is indeed not well visible in a single plane or small z projected depiction in the panels with suffix ii). However, this is well visible in the stacks that we had provided for the respective panels on FigShare. We hope that going through the stacks will convince you – the respective links are now given in the figure legends.

2) The GFP signal, which was taken from the same brain in another colour (respective panels with suffix i) supports our interpretation. We just did not want to have outlines in these more important panels.

We have addressed this issue in two ways: First, we removed half of the outlines in the respective figures, which allows the readers to judge by themselves. Second, we provide links to the respective original files on FigShare - both in the LSM and avi file formats (before, the data was available but not specifically linked for the respective figures).

3. The number of neurons innervating the CX that are Rx+ is not mentioned. Nor is the number of nearby neurons that are Rx+. How specific is this antibody/TF to the CX?

It is important to note that this line/antibody is not specific for cells that contribute to the CX – there are positive cells that project elsewhere. That illustrates why it is important to use additional anatomical criteria (cell body location and projection pattern) to homologize genetic neural lineages, as we have done. We have stressed the limits of the rx genetic neural lineage more clearly in the text (lines 185-189):

> *"Rx-positive cell groups likely belonged to eleven neural lineages projecting into many regions of the brain including central complex, mushroom bodies and other structures. Four lineages (DM1-4) were prominently marked in the imaging lines of both species. Because these lineages are known to contribute to the central complex we subsequently focused on the comparison of Rx-positive cell clusters of DM1-4."*

4. I don't understand the sequence heterochrony. The way it was introduced, it sounded like same events in fly and beetle occurred in a different order. But the data to me shows that the same events occur in the same order, but at different times. This is not surprising, and is not a novel finding.

We maintain that our data reveals a switch in the order of events – but apparently, we have done a poor job in visualizing this convincingly. We have prepared an additional Figure 11 to visualize this better than in the rather abstract scheme in Fig.10.

In this new figure, each scheme is redrawn from real data from figures of the results section such that the reader will be able to relate scheme to real data.

The essential switch of developmental events is the following: In *Tribolium* (right) steps 5-8 occur before the expansion of the neuropil during the larval period (step 4). We consider this expansion to be a distinct, important and homologous developmental step because it involves proliferation of neurons and a massive increase of axons that project into the CX anlagen leading to a strongly enlarged CX anlage. In *Drosophila*, these steps 5-8 occur after this phase of proliferation and neurite outgrowth.

5. The use of the term "functional" for neurons with pre-synaptic markers is inappropriate. It is taking an anatomical finding and extrapolating to a functional conclusion. I strongly feel that the term functional should be reserved for experiments that show a neuron activates or inhibits a downstream neuron (by ephys or optogenetics), or that a neuron can be stimulated by upstream neurons. Neither are shown here, so maybe swap out "functional" for "mature"?

In the semantic interpretation of the wording, the reviewer is correct. However: 1) We show that synapsin is active in that region and, importantly, in a previous paper, we showed that several neuromodulators are expressed there (serotonin, myo-inhibitory protein MIP, allatotropin, tachykinin-related peptide) (Koniszewski et al. 2016) – we regard it as reasonable to believe that this structure is functional. 2) Dr. Hartenstein is a very prominent figure in the neurodevelopment field and in our discussions he agreed that synapsin can be used as a proxy for functionality. 3) In the literature, the larval CX of beetles has always been assumed to be functional. 4) In the entire neuroanatomical literature, neuropils are assumed to be functional when expressing synapsin and there is no/not much data that would indicate that this assumption is wrong.

Hence, we would like to remain with our interpretation but we have made the degree of certainty and the limits of this statement more clear in the text (lines 278-283):

> *"Of note, we interpret the presence of synapsin as an indication for functionality, which in case of the Tribolium larval CX is supported by expression of several neuromodulators in specific patterns (9). However, an unequivocal proof of functionality of particular cells would require EM examination of the brains and electrophysiological recordings. Actually, the Drosophila L1 connectome confirms that all DM1-4 cells contributing to the central complex indeed lack synapses at the L1 stage (12), i.e. they are most likely not functional."*

Minor comments

6. I think the authors should describe and cite prior work on Eyeless/Pax6, which shows conservation in eye (neural tissue) beyond different taxa. To my knowledge, Ey expression has been traced from embryo to adult in multiple animals and shows conserved expression.

Thank you for this hint and we have included respective citations in the discussion. We note that the works that we have seen were restricted to cell body labelling by ISH/IHH of transcription factors. We go one step further and add projection pattern to such comparisons. We have added following sentence into the first discussion paragraph (lines 508-512):

> *"Previous efforts have nicely illustrated conservation of certain developmental genes and the cell types and organs they are required for, such as the eye in case of Pax6/eyeless (71,72). Our approach extends the analysis of cell type homology to labelling whole neurons combining regulatory gene expression and projection patterns as arguments for homology of neural cells."*

71. Erclik T, Hartenstein V, McInnes RR, Lipshitz HD. Eye evolution at high resolution: the neuron as a unit of homology. Dev Biol. 2009 Aug 1;332(1):70–9.
72. Halder G, Callaerts P, Gehring WJ. New perspectives on eye evolution. Curr Opin Genet Dev. 1995 Oct;5:602–9.

7. Why use synapsin instead of nc82/Brp? What are the differences? This could be spelled out in the methods. So much insect neuroanatomy is done with Brp, so the switch should be explained. Perhaps as simple as nc82 does not recognize Brp in Tribolium?

Outside the *Drosophila* field, the widely cross-reacting synapsin antibody has been used for many different insect species and is a well established marker for neuropil. Actually, we have tried many *Drosophila* antibodies in *Tribolium* and found that most of them do – unfortunately – not cross-react (see Koniszewski et al. 2016). Brp was not tested, because most antibodies do not cross-react and synapsin worked so well and it had been used in all other insects (several other beetle species, ants, etc).

8. Figure legends could be shortened to focus on describing the panel labels, which would reduce redundancy with the text.

We prefer that the main findings are given in the legends as well. Admittedly, this creates some redundancy. On the other hand, the hurried reader will be thankful for finding the main message in the legends as well.
In case of figure 1 we removed the redundant description from the text to the figure such that the knowing reader is not bored but the one that wants a reminder can refer to the figure. We made efforts to shorten the test of the other legends as well – with varied success.

Rev. 2:

General

The manuscript by Farnworth et al is an impressive piece of work that thoroughly compares the neural development of the central complex between the beetle Tribolium and the fly Drosophila. While much work has been done on this brain region in the fly, as well as in locusts, no means of homologizing neural lineages between species (beyond spatial location of cell bodies) existed so far. This paper uses the expression pattern of a conserved transcription factor to mark homologs neurons, and thereby labeling the offspring of four neuroblasts in the central insect brain. These neuroblasts give rise to the columnar cells of the central complex, a brain region that has shifted into the center of attention over the last decade and which is currently seen as the central decision making center of the insect brain. Defining core principles (extracted using a comparative approach) of how these regions develops is therefore of significant interest to the field. Moreover, the larvae of flies and beetles differ dramatically from each other with respect to their behavioral abilities, which correlates with the existence of a defined central complex. The authors of the current paper illuminate the exact timeline of the development of these regions, revealing significant shifts in timing of specific developmental events between flies and beetles, as well as shifts in the order of some of those events. Importantly, the immature central complex of the beetle appears to gain functionality already during the larval stage. During that stage, the larval central complex resembles the central complex of crustaceans more than that of the adult beetle, tempting the authors to speculate about phylogeny being reflected by ontogeny during insect central complex development.

The paper makes several main claims:
1) The concept of genetic neural lineages can be used to study homologous lineages across species.
2) Developmental steps within homologous neurons occur at different times and in some cases in a different order between flies and beetles, with the sequence change being shown for the first time in brain development.
3) The Tribolium central complex gains functionality at a developmental immature state.

The paper presents a wealth of data, the anatomical work has been carried out meticulously and is of high quality. The paper is framed as a developmental study, addressing comparatively specialized questions of neural development. Not being a developmental biologist by training, I found the paper relatively narrowly focused and rather technical in its language. This makes me a little bit doubtful if a broad life science audience is the best target group for this work. The authors might want to emphasize aspects of the work that are of high relevance to researchers beyond developmental biology in a revised version.

The main point that caught my attention is the proposed difference in onset of functionality of the central complex in beetles and flies. If this can be linked to behavioral abilities and the effective neural circuitry can be extracted from the Tribolium larval central complex, this would indeed be a significant interest to a wide audience, as it would establish a simplified model of a core decision making center of a brain. Of course, this cannot be achieved by a single paper; however, I would suggest that the authors rework the paper to highlight those broadly relevant aspects and condense the writing to a more streamlined story. If this is achieved and the points below are addressed, I think this paper would be very well suited for PLOS Biology.

Major comments

1) The introduction is very long and almost a review paper on its own. Please condense to guide the reader to what is the main story. Is the main question that of evolution of insects, or a question of novel, general principles of brain development, or a question of gaining a novel approach towards functionally understanding the central complex? The paper could be any of these (without changing the data), but the framing as it is leaves the reader a bit unsure about where the story will go.

Thanks for this feedback – we have shortened the introduction by 30% and tried to focus on heterochrony as one of the mechanisms of brain evolution. As we have substantially changed a lot of parts of the introduction, we have allowed ourselves to not track changes in this case, for the sake of readability. Overall, we have shortened the text (incl. refs) from 14.000 to 12.600 words

2) Functionality of the central complex is merely implied by the presence of synapsin labeling. Given that this is one of the major claims of the paper, I find this single approach not conclusive enough. At least one independent way of verifying that the larval CX of Tribolium is indeed functional, while that of Drosophila is not, would be needed to make this convincing. One could use EM imaging showing synaptic active zones, look at the expression of transmitters/receptors or voltage gated calcium channels (immunohistochemistry), or, optimally (but likely beyond reasonable scope for this paper), perform a conditional silencing experiment of the marked cells during the relevant life-stage and observe behavioral defects.

Indeed, our statement does not only rest on the presence of synapsin – in a previous work we had shown that a number of neuromodulators are expressed in the structure at the L1 stage. Hence, we feel that the additional requirement is already met. We have formulated this more explicitly in the text and explicitly added the limitation of our functionality interpretation (lines 278-283):

> *"Of note, we interpret the presence of synapsin as an indication for functionality, which in case of the Tribolium larval CX is supported by expression of several neuromodulators in specific patterns (9). However, an unequivocal proof of functionality of particular cells would require EM examination of the brains and electrophysiological recordings. Actually, the Drosophila L1 connectome confirms that all DM1-4 cells contributing to the central complex indeed lack synapses at the L1 stage (12), i.e. they are most likely not functional."*

Another problem of the Synapsin approach is also that there is a high chance of false negatives, i.e. even in Drosophila there might be a few synapses present (and active) that are simply below the detection ability of the immunohistochemistry. The problem then would be that the Drosophila CX might also be functional and the sequence heterochrony claim would become very shaky. Could the authors not explore the existing larval connectome of Drosophila and extract at least some examples of the neurons in the relevant lineages to show that there are no obvious active zones?

Thank you for this very important suggestion. We have done three-fold. First, we have re-checked the analysis done in Andrade et al. 2019 (https://doi.org/10.1016/j.cub.2018.12.012). Here, the Hartenstein lab used the larval connectome to identify a population of small undifferentiated neurons. Interestingly, ALL midline-crossing cells that belong to the DM1-4 lineage group (i.e. those that we homologized in this work) indeed lack synapses. Second, we have had extensive personal communication with Prof. Hartenstein where he stressed that his intense work on the CX of Drosophila did not lead to any indication of functionality. He and us are not aware of any potential cells that could contribute to the larval CX and provide synapses.
Third, we have highlighted the Andrade et al 2019 paper in the text at lines 282-283:

> *"Actually, the Drosophila L1 connectome confirms that all DM1-4 cells contributing to the central complex indeed lack synapses at the L1 stage (12), i.e. they are most likely not functional."*

3) The authors state that the Tribolium genetic labelling only marks 5-10% of their genetic neural lineage, while in Drosophila all cells are marked. This raises some doubt as to how meaningful the comparison is. This is a problem when specifics of arborization patterns are discussed. As the claim of homology of the studied cells is at the core of the paper, this seems problematic. That said, I am unsure how serious this is, and the authors should address this issue even more clearly than they already do. What could we be missing? What are the 95-90% of the cells in the lineage that we are

not seeing? What evidence is there that they follow the same principles as the ones that are labelled? See also point 5 in minor comments.

We agree that it is very unfortunate that this enhancer trap marks a subset only and of course, we have been trying to generate a respective line in Tribolium (more difficult than expected…). We deal with this issue in two ways: First, we quantified the overlap and found that in earlier stages the overlap is much larger. Second, we argue that this issue does not interfere with the claims and interpretations.

1. We added quantifications of the fraction of double-positive *rx*/GFP cells in the region of DM1-4 cells at three different stages (adult, prepupa, L1) by counting. The results show that indeed in the adult the fraction is approximately 10 %. However, in the prepupa is already around 50 % and in L1 it is 75 %. Hence, we are confident that our interpretations reflect the actual situation, within the limitations of this transgenic line.

2. a) In this work we focus on a subset of cells, i.e. those that have similar cell body location and projection patterns into the CX in the adult brain. Based on these similarities at the endpoint of development, we state their homology. Then we compare the development of this subset of cells (and only of these). There are non-marked cells and there could be marked non-homologous cells in the Tribolium enhancer trap line – but as long as we focus on the marked homologous cells these potential other cell groups do not interfere with our analysis.
- One key statement is the reduced projection pattern of these cells in Drosophila larval brains. To make this point, it was indeed essential that all cells are marked in this line such that we can be confident that we are not arguing based on false negative data.
- We cannot exclude that the real number of cells in the homologous cell group in Tribolium might be higher than the marked portion of homologous cells. This uncertainty made us cautious with quantifying and giving numbers of cells in both species.

Changes to manuscript and SI:
- Quantification data is shown in the Fig. S2
- the numbers are given in the text (lines 152-155)

> *"The enhancer trap marked a subset of Tc-Rx-positive cells in the region of the central complex depending on the stage (77.39 % in L1, 50.23 % in the prepupa, 9.71 % in the adult; Fig. S2). Importantly, all EGFP-positive cells were Tc-Rx-positive as well (Fig. S2)."*

Discussion (lines 534-541):
"The fact that our Tribolium enhancer trap line did not mark all Tc-Rx positive cells does fortunately not interfere with our conclusions. First, at developmental stages 50-75 % of the cells were marked while only in the adult the portion dropped to 10%. Second, we focus on marked cells that are homologous between both species. As we restrict our statements on this marked subset of cells, the presence of non-marked cells in Tribolium does not interfere with our interpretation. Third, only in Drosophila, we make a statement on the lack of projection at a certain stage. Luckily, the Drosophila bicistronic line showed a 100% overlap of EGFP with Dm-Rx expression allowing us to make this statement."

Supporting Information (lines 385-391):
"Manual quantification was performed by first limiting image data to the region of interest in all three dimensions (Fig. S2Fi). Then, background was removed by modifying brightness and contrast. Then, we determined how many slices approximately covered one cell and made small Z projections. Using the Cell Counter plugin of ImageJ, we counted the number of cells in the projection. We exported the markers and then we overlapped these markers with the next progressive Z projection to avoid counting any cell twice. Cells were counted, means and standard deviations calculated for N=3 brains."

4) What is a behavioral relevance of a functional CX in the larvae? What can the beetle larvae do that the fly larvae cannot. While I would of course be happy to see this addressed experimentally, I think that this might be better left for future studies. However, I believe the authors should provide a roadmap towards addressing what their findings mean for the neuroethology of the beetle larva as well as review what is known about their behavioral differences. In my view, this would be essential to make the paper more relevant to a broad audience.

We agree that this would be extremely interesting but we consider it also extremely challenging, to quantify the differences in behavior between species. It is already quite a task to set up paradigms to quantify differences in behavior within a species. But how to compare the behavior of a legless larva with highly reduced eyes being submerged in humid food with a larva that has legs, eyes and burrows in very dry conditions?
We are planning to answer this question by teaming up with behavioral experts on the Göttingen Campus. But this will be a full project on its own – and a challenging one.
Unfortunately, there is not much to review because to the best of our knowledge, there are no respective behavioral comparisons.
Therefore, we have expanded the respective discussion with speculations and present an experimental way for testing this. Please find this under the heading (line 626) *“Behavioral implications – An immature but functional central complex”*

5) The idea that the larval CX is a regression towards a more ancient form of the CX found in crustaceans seems a bit too speculative. Sure, they look similar, but is there any other piece of evidence that points in the direction, e.g. single neuron morphologies, immunostaining patterns, EM data? Also, the crayfish is obviously not the ancestor of any insect, and claims about how the common ancestor's CX might have looked must be backed at least by explicitly stating the data on which those assumptions are based. to me the paper is interesting without those speculations and I suggest to leave them out.

We have to agree that this was a bit far fetched and we followed the suggestion to remove this part.

6) The authors spend significant effort to establish their genetic methods that allow them to observe homologous lineages between flies and beetles. They state that their methods provides a unique way to study the development of the CX in a comparative way. Yet, in the discussion, they present data on the locust (based on George Boyan's work) that provides very similar data, but without having these methods available. The stereotypical position of the neuroblasts and the highly conserved projection patterns of the CX columnar cells appear to therefore render the genetic lineage concept almost unnecessary. This might not be the case for other lineages, which give rise to more distributed sets of neurons, but the advantages of the genetic lineage concept would need to be pointed out in a more explicit way.

There are some aspects that require a genetic marking of a subset of homologous cells, while some aspects of this work could indeed have been done with “classical” stainings.
We added an entire paragraph on this under the heading: “Genetic neural lineages as a tool for evolutionary neural development”. In addition:

- Only the marking of homologous cells of two species allows reliably tracing back comparable cells from the adult to the embryo in two species. Other stains like acetylated tubulin or others do reflect the overall heterochrony but they do not allow any statement on the differences in development of homologous cells - just because all cells are marked. For instance: When comparing the developmental time of formation of the first commissure based on acetylated tubulin – are we sure that this event is performed by homologous neurons? In contrast, seeing the first midline crossing by marked neurons of a genetic neural

lineage allows to exactly determine the time when a homologous developmental stage of homologous cells occurs. This is the precision we are aiming for.
- Indeed, thorough insights have been gained by DiI labelling in Schistocerca. However, this technique is difficult to transfer to small insects – when last I talked to Dr. Urbach, he mentioned that he did not manage to label homologous neuroblasts in the Drosophila brain apart from the large MB neuroblasts (while the Technau lab where he was working at the time had been very successful doing this with the ventral nerve cord).
- While DiI labelling is an interesting option for large embryos, we need the work on a beetle because they are the best developed genetic model systems that show this intermediate heterochrony between Drosophila and hemimetabolous insects. In the end we want to identify the genetic underpinnings as well, for which we need to work on a genetic model (which turns out to be rather small).

Minor comments

1. Figure 1: Add tangential cells of the noduli to complete the set of cell types.

Done. We added this citation as source:
23. Hadeln J, Hensgen R, Bockhorst T, Rosner R, Heidasch R, Pegel U, et al. Neuroarchitecture of the central complex of the desert locust: Tangential neurons. Journal of Comparative Neurology [Internet]. 2019 Oct 18 [cited 2019 Nov 4]; Available from: https://onlinelibrary.wiley.com/doi/abs/10.1002/cne.24796

2. Nomenclature: I understand that the authors want to use the CBL/CBU nomenclature that was used in locusts and other classic insect model species. However, the Drosophila EB/FB naming scheme is much more widespread by now, is the official recommendation by the brain name group (Ito et al. 2014) and both systems make equally no sense in many species. Given that the paper compares Tribolium with Drosophila, the authors would prevent a lot of confusion, if they change the paper to the EB/FB naming scheme (including the other changes that come with this system). Additionally, the developmental work on locusts by Boyan et al., has used the terms EB and FB anyway, making those comparisons easier as well.

Done.

3. Line 313: The non visibility of columnar division in the larval central body is presented as distinctive from the adult. Yet, the images in Figure 3 do not show any evidence for columns either (as in many insects). This should be rephrased somehow.

We have removed this statement. We now state columnar organization for the first time for the larval stage (Fig. 6).

4. Line 333 and following: The authors argue that the decussations found in the larva based on tubular staining are less prominent than those visible via EGFP in the pupa. This might be true, but GFP expressing fibers are likely more visible compared to fibers that are labeled by immunohistochemistry, even if their numbers are the same. Is there any quantitative information that the authors could provide (numbers of fibers)?

Thank you - We have not done respective quantifications and have removed this statement.

5. Line 378 and following: The authors use labelled cells in both species to reveal the timing of certain events during development, and find that the Drosophila CX develops faster in the beginning. However, the cells labelled in Tribolium are only a small subset of those labelled in the fly. What if only the neurons born late in the lineage express EGFP?Do you have data on where

within the lineage the labelled cells are located? Do they include the first cells as well, which pioneer the midline crossings? If not, then how can the authors be sure their timing comparison is valid?
(see our reply to your major comment 3) We agree that we cannot be entirely sure. However, we noted that the overlap of marked neurons with the enhancer trap is much higher at the end of embryonic development (~ 75 %) compared to the adult (~ 10 %). We have added these data in the revised version.

Hence, we assume that over 50% of cells are labelled at the mentioned stage and that our assumptions are likely correct. Importantly, the respective statement does not touch our key finding, which is the sequence heterochrony.

This question could only be answered with a bicistronic line in Tribolium, which we have tried to do in the course of the project but so far did not got lines with the expected pattern. Unfortunately, this approach targeting an essential gene is not well established in Tribolium and we are working on it.

6. Lines 388 and following: The final larval pattern (including a distinct PB as argued earlier and shown in Fig. 4) is reached at 81% of embryonic development. But the authors then say that no distinct PB could be detected in the embryo. This seems a contradiction, or is the latter sentence referring to Drosophila?

Indeed, in late embryonic stages we see only a diffuse pattern of synapsin, hence no clearly delineated PB. This pattern develops between this late stage embryo and hatching, which is why in Fig. 10 the steps of functional CB and PB are not shown at the embryonic but on L1 stage. We have modified the text and hope that the confusion is cleared (lines 337-339):

> *"A distinct protocerebral bridge or central body that was clearly differentiated from other areas was not detectable in the embryo, neither in Tribolium nor Drosophila."*

7. Line 422: Add 'of larval development' after 50% to be clear (after the previous section talked about embryonic development)

Done.

8. Line 440: a qualitative increase of cell number is a very odd phrase. Numbers are quantitative by definition. Please rephrase or count the cells. But given the subset labeling issue mentioned above, what do these numbers mean in Tribolium?

We rephrased as suggested: *"In both species' larval brains, we observed an increase in cell number of the DM1-4 rx-positive cell bodies (not quantified)"*

9. Fig. 6: The PB in panel B seems equally structured to me than that of panel F. Stating that columns emerge during larval development in the PB seems a bit at odds with at least the shown images. Ideally, one would want to quantify the staining distribution along the PB to get more solid data on this.

We do not see any columnar organization in L1 (Fig. 4F^{i}) but we do see it quite clearly in late larvae (Fig. 6F^{ii}). We found it difficult to quantify given the complex and differing position in 3D in the different stages. Hence, we re-worded such that we refrain from strong statements (lines 380-382):

> *"The columnar architecture of the protocerebral bridge appeared to develop during larval stages. While we did not see columns in L1 (Fig. 4F^{ii}) we do see them more clearly in late larval stages (Fig. 6F^{ii}) and probably already in 50% larvae (Fig. 6B^{ii})."*

10. Line 543 and following: Could it not also be that the rx positive cells do the early switch, but are not included in the 5-10% subset that is labeled by the EGFP expression? Or am I misunderstanding what the EGFP line shows?

In the pupa, a larger portion of cells is marked in the enhancer trap line (see above). Hence, we regard this as an unlikely scenario. However, to be sure, we have removed this statement as it is not necessary for our conclusions.

11. Line 570: is there not a simpler word for 'paedomorphocline'? Addressing a broad audience would benefit from changing highly specialized wording for simpler terms.

We use “juvenilization” and add “paedomorphocline” in brackets for the heterochrony aficionados.

12. Figure 10: Please include a legend in the panels to guide the reader.

We added a legend to the panels.

13. Line 667/668: this is a very fuzzy statement. More details would be needed to illustrate potential effects of the presence of a functional CX in the larva.

The statement was deleted during the re-writing of the discussion.

14. Line 666: a 'prominent' columnar architecture is not visible in most insects, unless one looks at single neurons. Thus, it should not be used as a distinguishing feature if nothing but synapsin staining is used to judge the existence of columns.

We agree, have removed this phrase, and checked the rest of the manuscript for this or similar phrasing.

15. section starting in line 670: I find the entire section not very clear and I have a hard time following the argument.

We tried to make the section more clear.
