# Supplementary material for: Sequence heterochrony led to a gain of functionality in an immature stage of the central complex: A fly–beetle insight
Source: PLoS Biol. 2020 Oct 26;18(10):e3000881. doi: 10.1371/journal.pbio.3000881 (PMC7644108; doi:10.1371/journal.pbio.3000881)
Supplement: Attachment — Submitted filename: Response to reviewers comments final revision 2.docx [file pbio.3000881.s018.docx]

Changes suggested by reviewer 2:

**Line 226: please explain what SME means in the figure legend**

*… are smooth manifold extractions (SME)s (66)…*

**Line 320: Did the last sentence of the previous paragraph not answer the question posted here? This section investigates this issue in much more detail and more thoroughly, so nothing is wrong with the content. Please rephrase to avoid the apparent contradiction of asking an already answered question.**

We tried to make more clear the distinction from the previous paragraph:

*Given the heterochronic state found in the L1 larva we asked, how this difference developed during embryogenesis. Specifically, we wanted to test, whether the observed differences were due to by simple temporal shifts within a conserved developmental series (i.e. classic heterochronic shift) or whether certain steps of the developmental series had flipped their position within the series (i.e. sequence heterochrony).*

**Line 442: I don't see how the change in shape corroborates that the CX in larvae is immature but functional. Also, the sentence is quite repetitive, so I suggest to leave out the half sentence after 'corroborating'.**

Deleted as suggested

**Line 567: Please specify the species of bee, I assume honeybee, but maybe be more precise.**

Indeed honey bee:

*… castes of the honey bee Apis mellifera, where …*

**Line 694: naming statement regarding CBL and CBU: I think this sentence has become obsolete, as now, EB and FB are used.**

Good point. We modified the sentence to explain our use of the nomenclature:

*We refer to the divisions of the central body as fan-shaped and ellipsoid bodies for sake of comparability with Drosophila literature. Where morphologically meaningful, we use the synonyms upper and lower division, which are terms used in classic insect literature.*
